# Manumycin inhibits *ras* signal transduction pathway and induces apoptosis in COLO320-DM human colon tumourcells

**DOI:** 10.1054/bjoc.1999.1018

**Published:** 2000-02

**Authors:** A Di Paolo, R Danesi, D Nardini, G Bocci, F Innocenti, S Fogli, S Barachini, A Marchetti, G Bevilacqua, M Del Tacca

**Affiliations:** Division of Pharmacology and Chemotherapy and Division of Pathology, Department of Oncology, University of Pisa, Via Roma 55, Pisa, I-56126, Italy

**Keywords:** manumycin, colorectal neoplasms, *ras* proteins, apoptosis, protein isoprenylation

## Abstract

The aim of the present study was to assess the cytotoxicity of manumycin, a specific inhibitor of farnesyl:protein transferase, as well as its effects on protein isoprenylation and kinase-dependent signal transduction in COLO320-DM human colon adenocarcinoma which harbours a wild-type K- *ras* gene. Immunoblot analysis of isolated cell membranes and total cellular lysates of COLO320-DM cells demonstrated that manumycin dose-dependently reduced p21 *ras* farnesylation with a 50% inhibitory concentration (IC_50_) of 2.51 ± 0.11 μM and 2.68 ± 0.20 μM, respectively, while the geranylgeranylation of p21 *rhoA* and p21 *rap1* was not affected. Manumycin dose-dependently inhibited (IC_50_= 2.40 ± 0.67 μM) the phosphorylation of the mitogen-activated protein kinase/extracellular-regulated kinase 2 (p42MAPK/ERK2), the main cytoplasmic effector of p21 *ras*, as well as COLO320-DM cell growth (IC_50_= 3.58 ± 0.27 μM) without affecting the biosynthesis of cholesterol. Mevalonic acid (MVA, 100 μM), a substrate of the isoprenoid synthesis, was unable to protect COLO320-DM cells from manumycin cytotoxicity. Finally, manumycin 1–25 μM for 24–72 h induced oligonucleosomal fragmentation in a dose- and time-dependent manner and MVA did not protect COLO320-DM cells from undergoing DNA cleavage. The present findings indicate that the inhibition of p21 *ras* processing and signal transduction by manumycin is associated with marked inhibition of cell proliferation and apoptosis in colon cancer cells and the effect on cell growth does not require the presence of a mutated *ras* gene for maximal expression of chemotherapeutic activity. © 2000 Cancer Research Campaign


*British Journal of Cancer* (2000) **82**(4), 905–912
© 2000 Cancer Research Campaign
DOI: 10.1054/ bjoc.1999.1018, available online at http://www.idealibrary.com on IDEAL®

### Manumycin inhibits *ras* signal transduction pathway and induces apoptosis in COLO320-DM human colon tumour cells

**A Di Paolo[1], R Danesi[1], D Nardini[1], G Bocci[1], F Innocenti[1], S Fogli[1], S Barachini[1], A Marchetti[2], G Bevilacqua[2] and M Del Tacca[1]**

[1]Division of Pharmacology and Chemotherapy and [2]Division of Pathology, Department of Oncology, University of Pisa, Via Roma 55, I-56126 Pisa, Italy

**Summary** The aim of the present study was to assess the cytotoxicity of manumycin, a specific inhibitor of farnesyl:protein transferase, as well as its effects on protein isoprenylation and kinase-dependent signal transduction in COLO320-DM human colon adenocarcinoma which harbours a wild-type K-*ras* gene. Immunoblot analysis of isolated cell membranes and total cellular lysates of COLO320-DM cells demonstrated that manumycin dose-dependently reduced p21*ras* farnesylation with a 50% inhibitory concentration ($IC_{50}$) of 2.51 ± 0.11 μM and 2.68 ± 0.20 μM, respectively, while the geranylgeranylation of p21*rhoA* and p21*rap1* was not affected. Manumycin dose-dependently inhibited ($IC_{50}$ = 2.40 ± 0.67 μM) the phosphorylation of the mitogen-activated protein kinase/extracellular-regulated kinase 2 (p42MAPK/ERK2), the main cytoplasmic effector of p21*ras*, as well as COLO320-DM cell growth ($IC_{50}$ = 3.58 ± 0.27 μM) without affecting the biosynthesis of cholesterol. Mevalonic acid (MVA, 100 μM), a substrate of the isoprenoid synthesis, was unable to protect COLO320-DM cells from manumycin cytotoxicity. Finally, manumycin 1–25 μM for 24–72 h induced oligonucleosomal fragmentation in a dose- and time-dependent manner and MVA did not protect COLO320-DM cells from undergoing DNA cleavage. The present findings indicate that the inhibition of p21*ras* processing and signal transduction by manumycin is associated with marked inhibition of cell proliferation and apoptosis in colon cancer cells and the effect on cell growth does not require the presence of a mutated *ras* gene for maximal expression of chemotherapeutic activity. © 2000 Cancer Research Campaign

**Keywords:** manumycin; colorectal neoplasms; *ras* proteins; apoptosis; protein isoprenylation

During the last years, several studies have demonstrated that *ras* genes are involved in the signal transduction from transmembrane receptors to intracellular effectors, thus controlling cell differentiation and growth (Marshall, 1996). Alteration of the normal biologic function of *ras* proteins might be associated with the acquisition of a neoplastic phenotype of cells (Lowy and Willumsen, 1993). Mutational activation of *ras* oncogenes is present in up to 90% of pancreatic cancers and 50% of all cases of colorectal cancer (Bos, 1989), two neoplasms poorly responsive to the treatment with traditional chemotherapeutic agents. Moreover, the mutational activation of *ras* is associated with a poor prognosis and a shorter survival of patients (Rodenhuis et al, 1997). Thus, the inhibition of *ras* protein function could be an effective pharmacological approach in cancer chemotherapy.

Proteins belonging to the *ras* superfamily exert their functions when they are localized to specific cellular compartments, and this targeting is made possible by post-translational modifications (Marshall, 1993). The first of them consists of the farnesylation of the cysteine residue at the carboxyterminal tetrapeptide CAAX, where C is cysteine, A any aliphatic amino acid, and X methionine or serine (Maltese, 1990). This step is crucial, because its inhibition causes the interruption of the subsequent processing of the cytosolic immature *ras* proteins, thus impairing their biological functions (Gibbs, 1994). In this regard, numerous attempts have been directed to the identification of chemotherapeutic agents inhibitors of *ras* farnesylation (Tamanoi, 1993; Levitzky, 1996). Among them, manumycin is a promising compound derived from *Streptomyces parvulus* and characterized by Hara et al (1993). Manumycin acts via the specific inhibition of the farnesyl:protein transferase (FPTase), as demonstrated by the reversion of a *ras*-dependent phenotype in the worm *Caenorhabditis elegans* (Hara and Han, 1995) and exerts antiproliferative effect on human tumour cells which harbour a mutated K-*ras* gene (Nagase et al, 1996). However, the issue of therapeutic targeting of normal *ras* proteins has not been addressed and data are lacking concerning the ability of FPTase inhibitors to suppress the proliferation of tumour cells with wild-type K-*ras* genes.

In the present work, the effect of manumycin was evaluated on the processing of isoprenylated proteins of the *ras* superfamily, *ras* signal transduction pathway and cell death in the cell line COLO320-DM derived from a human colorectal carcinoma.

*Received 25 November 1998*
*Revised 5 August 1999*
*Accepted 25 August 1999*

*Correspondence to:* M Del Tacca

### METHODS

### Drugs and chemicals

Antipain, leupeptin, aprotinin, sodium dodecyl sulphate (SDS) and proteinase K were purchased from Boehringer Mannheim GmbH (Mannheim, Germany). RPMI-1640 and fetal bovine serum (FBS) were from HyClone (Cramlington, UK); L-glutamine, agarose and 180-bp DNA ladder were purchased from Gibco (Gaithersburg,

MD. USA) and acrylamide was from International Biotechnologies Inc. (New Haven, CT, USA). Diethylamine (DEA) and Nonidet P-40 were obtained from ICN Biomedicals Inc. (Costa Mesa, CA, USA); mouse IgG1 anti-p21*ras* monoclonal antibody was purchased from Transduction Laboratories (Lexington, KY), while anti-p21*rap1*, anti-p21*rhoA* and anti-mitogen-activated protein kinase/extracellular-regulated kinase 2 (p42MAPK/ERK2) rabbit polyclonal antibodies were from Santa Cruz Biotechnology Inc. (Santa Cruz, CA, USA). Horseradish peroxidase-conjugated secondary antibodies (dilution 1:10 000) and reagents for chemiluminescent detection of protein immunoblots were purchased from Amersham Life Science (ECL Western detection kit, Little Chalfont, UK). All other chemicals not listed in this section were from Sigma Chemical Co. (St Louis, MO, USA).

Manumycin was a generous gift from Dr M Hara, Tokyo Research Laboratories (Tokyo, Japan); the drug was dissolved in sterile distilled water containing dimethyl sulphoxide (DMSO) 0.05% at 10-mM concentration, and stored at –20°C. Manumycin and mevalonic acid (MVA) were diluted in sterile culture medium immediately before their use.

### Cell culture

The human colorectal cancer cell line COLO320-DM was obtained from the American Type Culture Collection (ATCC, Rockville, MD, USA). COLO320-DM cells were maintained in RPMI-1640 medium, supplemented with 10% FBS, penicillin (50 IU $ml^{-1}$), streptomycin (50 μg $ml^{-1}$) and L-glutamine (2 mM). Cells were grown in 75-$cm^2$ tissue culture flasks (Nunc, Roskilde, Denmark) and kept in a humidified atmosphere of 5% carbon dioxide ($CO_2$) at 37°C. Cells were harvested with a solution of trypsin–EDTA when they were in log phase of growth, and maintained at the above-described culture conditions for all experiments.

### Polymerase chain reaction analysis of K-*ras* sequence in COLO320-DM cell line

In order to demonstrate the possible mutations of K-*ras* sequence, the mutational analysis of the codon 12 of the K-*ras* gene was performed in COLO320-DM cells by oligodeoxynucleotide hybridization, according to the method previously described (Marchetti et al, 1996). As a negative control, the CLONE cell line (ATCC, Rockville, MD, USA), derived from a normal human corneal epithelium was analysed, while as a positive control a sample of human lung adenocarcinoma with mutation at codon 12 (GGT → TGT, Gly → Cys) was used. The primers used to amplify the K-*ras* gene around codon 12 were: 5′-GGCCTGCTGAA-AATGACTGA-3′ and 5′-TGATTCTGAATTAGCTGTAT-3′. The polymerase chain reaction (PCR) was programmed as follows: initial denaturation, 4 min at 94°C; amplification, 30 s at 94°C, 30 s at 54°C and 1 min at 72°C for 35 cycles; elongation, 10 min at 72°C. The amplified products of the PCR were denatured, blotted onto nylon membranes and then hybridized with $^{32}P$-labelled oligonucleotide probes specifically designed to detect *ras* mutations (Marchetti et al, 1996).

### Immunoblot analysis of p21*ras*, p21*rap1*, p21*rhoA* and p42MAPK/ERK2

In order to document the farnesylation of p21*ras* and its association with the cell membrane, COLO320-DM cells were exposed to manumycin 1–10 μM for 24 h and membranes were separated according to the method of Nagasu et al (1995), with minor modifications. Briefly, cells exposed to manumycin and untreated controls were harvested with ethylenediaminetetraacetic acid (EDTA), washed three times with phosphate-buffered saline (PBS), and centrifuged at 1000 rpm at 4°C for 5 min. Cells were finally resuspended in 5 mM EDTA, 5 mM Tris-base (pH 7.6), 1 mM phenylmethylsulphonyl fluoride (PMSF), sonicated four times for 10 s, and centrifuged at 12 000 rpm for 5 min at 4°C. The supernatant was then centrifuged at 33 500 rpm for 30 min at 4°C, and the resulting membrane pellet was solubilized in 0.01% sodium dodecyl sulphate (SDS). Protein concentration of samples was measured according to the method of Lowry et al (1951). Proteins were then separated on 11% SDS-polyacrylamide electrophoresis gel (SDS-PAGE), and transferred onto Immobilon-P membrane (Millipore, Bedford, MA, USA) by using a Multiphor II NovaBlot cell (Pharmacia, Uppsala, Sweden). Blots were probed with an anti-p21*ras* antibody (1:1000) and detected with the use of horseradish peroxidase-conjugated secondary antibody (dilution, 1:10 000). The membranes were then exposed to a Kodak X-Omat AR film, and film densities were quantified as described in the section Analysis of Data.

In order to evaluate whether the reduction in membrane-bound farnesylated p21*ras* is associated with the increase in the immature, non-farnesylated *ras* in the cytoplasm, total cellular extracts were analysed for p21*ras* by immunoblotting. Briefly, cells exposed to manumycin 1–10 μM for 24 h and untreated controls were harvested with EDTA, washed three times with PBS and centrifuged at 10 000 rpm for 5 min at 4°C. The cells were solubilized at 4°C in Tris-base 50 mM pH 7.6, 2 mM EDTA, 100 mM sodium chloride (NaCl), 1% Nonidet, 1 μM PMSF and 2 μg $ml^{-1}$ each of aprotinin, pepstatin and antipain for 45 min. Cell lysate was then centrifuged at 15 000 rpm for 20 min, and the pellet was discarded. Aliquots of supernatants were used to measure protein concentration as described above, and the remaining samples were used for immunoblot assays. Samples were boiled for 5 min in SDS-sample buffer (50 mM Tris-base pH 6.8, 2% SDS, 100 mM dithiothreitol, 10% glycerol and 0.025% β-mercaptoethanol) and separated on 12.5% SDS-PAGE. Proteins were transferred onto Immobilon-P membrane, probed with anti-p21*ras* antibody (1:1000) and detected as described above.

To evaluate the effect of manumycin on protein geranylgeranylation and p42MAPK/ERK2 phosphorylation, COLO320-DM cells were treated with manumycin 1–25 μM for 24 h. Cells were solubilized in 1% Nonidet P-40, 50 mM Tris-base pH 7.6, 2 mM EDTA, 100 mM NaCl, 1 mM PMSF and 5 μg $ml^{-1}$ each of antipain, aprotinin, pepstatin for 45 min at 4°C. In the case of p42MAPK/ERK2 analysis, the protein phosphatase inhibitors sodium metavanadate and sodium fluoride (200 μM each) were added to the lysis buffer (Danesi et al, 1995). Cellular lysates were centrifuged for 20 min at 15 000 rpm, and aliquots of supernatants were separated to measure protein concentration.

Total cellular proteins (100–200 μg) were boiled for 5 min in SDS-sample buffer and separated on 11% (p42MAPK/ERK2) and 12.5% (p21*rhoA* and p21*rap1*) SDS-PAGE (Danesi et al, 1996). Proteins were transferred onto Immobilon-P membrane and probed with anti-p42MAPK/ERK2, anti-p21*rhoA* and anti-p21*rap1* antibodies (dilution, 1:1000), and detected as described above.

© 2000 Cancer Research Campaign

### Analysis of cellular cholesterol levels

In order to document whether manumycin was able to affect cholesterol synthesis, total cholesterol concentration was determined in $1.5 \times 10^6$ COLO320-DM cells treated with manumycin 1–10 μM for 24 h. Cells were harvested with trypsin-EDTA and lysed in 55 μl of Tris-EDTA (TE) buffer (8 mM Tris-base pH 7.6, 0.5 mM EDTA pH 7.4) containing 0.5% Triton X-100 for 75 min at 4°C. Aliquots of suspensions were used to measure protein concentration as described by Lowry et al (1951). Cholesterol was assayed using the Cholesterol Detection Kit (Sigma Chemical Co., St Louis, MO, USA), based on cholesterol esterase/oxidase enzymatic reactions, by measuring the absorbance at 500 nm of a peroxidic dye product. Cholesterol levels were normalized to the amount of protein in the sample and expressed as μg of cholesterol $mg^{-1}$ of protein.

### Assay of apoptosis by manumycin

To demonstrate whether manumycin was able to trigger apoptosis, COLO320-DM cells were treated with manumycin 1–25 μM for 72 h, alone or in combination with MVA 100 μM. Cells were harvested with trypsin–EDTA, and $3 \times 10^6$ cells for each sample were centrifuged at 2600 rpm for 5 min and processed as previously reported (Danesi et al, 1995). Briefly, cells were lysed in TE buffer for 90 min at 4°C; cellular lysates were centrifuged at 15 000 rpm for 1 h at 4°C, and clear supernatants containing fragmented chromatin were digested at 42°C for 30 min with proteinase K (200 μg $ml^{-1}$). Samples were then diluted 1:1 in phenol:chloroform:isoamyl alcohol, vigorously shaken for 30 s and centrifuged at 15 000 rpm for 10 min.

Supernatants were collected and mixed with 100 μl of NaCl 5 M, 1 ml of ethanol (4°C) and 1 μl of glycogen. The suspensions were kept at –20°C overnight to precipitate DNA fragments, then centrifuged at 15 000 rpm for 30 min; the supernatants were discarded and the pellets were washed with 70% ethanol and dried under air flow.

Each sample was resuspended in TE buffer containing 1 mg $ml^{-1}$ of boiled bovine pancreatic RNAase, incubated at 40°C for 1 h and then mixed with DNA sample buffer (15 mM EDTA pH 8.0, 0.1% SDS, 0.025% xylene cyanole, 0.025% bromophenol blue and 0.5% glycerol). Separation of DNA fragments was obtained by electrophoresis in 1% agarose gel, in Tris–EDTA–acetate (TAE) buffer (Tris-base 32 mM, 1% glacial acetic acid and 1 mM EDTA), and bands were visualized by ethidium bromide staining under UV light. Gel was photographed with a Polaroid MP4 Land camera (Polaroid, Cambridge, MA, USA) and pictures were digitized for the analysis of DNA fragmentation as described in the section Analysis of Data.

### Analysis of cytotoxicity

The effect of manumycin on COLO320-DM cell growth was evaluated on $1.5 \times 10^6$ cells $well^{-1}$ seeded in flat-bottomed 6-well plates (Nunk, Roskilde, Denmark), in a final volume of 1 ml $well^{-1}$, and incubated at 37°C in 5% $CO_2$. At day 2, cells were treated with manumycin 0.1–25 μM for 24 h, and then harvested with trypsin–EDTA to measure their number. To assess the possible effect of MVA on the antiproliferative activity of manumycin, COLO320-DM cells were exposed for 24 h to manumycin 0.1–25 μM in combination with MVA 100 μM, and cell survival was calculated as described above. Proliferation of treated cells was expressed as mean per cent values ± standard error (s.e.) with respect to untreated controls.

### Analysis of data

Film densities of protein immunoblots and apoptosis assays were quantified through video imaging densitometry with the Kontron Imaging system KS300 (Kontron Elektronic, Eching, Germany) connected to a TK-1280E colour video camera (JVC, Tokyo, Japan), and expressed as arbitrary units of mean grey values ± s.e. (Danesi et al, 1996). The degree of apoptosis was assessed either by single-band densitometric analysis of DNA fragments in the range of 180–900 bp as well as by calculating the fragmentation ratio (FR), as described by Kawabata et al (1994). Briefly, each lane of agarose gels was divided into three areas: L area, containing DNA fragments larger than 10 kbp; M area containing DNA from 10 kbp to 0.3 kbp; and S area, the nucleosomal size area, with DNA smaller than 0.18 kbp. The integration of the total area of each lane (A) as well as M and S areas were IA, IM and IS, respectively, and the FR was calculated as follows:

$$\mathrm{FR}(\%) = 100 \times (0.5 \times \mathrm{IM} + 1 \times \mathrm{IS})/\mathrm{IA}$$

The analysis of the effects of manumycin on p21*ras*, p42MAPK/ERK2 and cytotoxicity included the non-linear least squares curve fitting of the experimental data in order to calculate the drug concentration producing a 50% decrease in the optical densities and cell proliferation ($IC_{50}$). Results obtained from triplicate experiments are given as mean values ± s.e. The two-tailed unpaired Student's *t*-test was used to assess the statistical differences of data obtained in control and treated cells with respect to the analysis of protein immunoblots, cholesterol levels, extent of apoptosis and cytotoxicity. $P$ -values lower than 0.05 were considered significant.

### RESULTS

### Absence of K-*ras* gene mutation in COLO320-DM cell line

The determination of point mutations at codon 12 of K-*ras* oncogene was performed by PCR amplification of fragments surrounding codon 12 of K-*ras* using genomic DNA as a template. Amplified DNAs were hybridized with wild-type and mutation-specific oligonucleotide probes. The analysis of DNA extracted from COLO320-DM cells demonstrated indeed a normal K-*ras* gene sequence while a GGT→TGT mutation was evident in one allele of the lung tumour sample (Figure 1).

### Inhibition of p21*ras* farnesylation and p42MAPK/ERK2 phosphorylation by manumycin

Immunoblots of COLO320-DM cell membranes or total cellular lysates probed with anti-p21*ras* antibody revealed that manumycin inhibited the post-translational processing of immature p21*ras*, because increasing drug concentrations caused a proportional decrease in the amount of farnesylated p21*ras* (Figure 2A and C). Indeed, image analysis of signals obtained after Western blot assay of p21*ras* associated to cell membranes confirmed that manumycin reduced the amount of membrane-bound protein (Figure 2B), that is, the amount of farnesylated p21*ras*, and the

© 2000 Cancer Research Campaign 

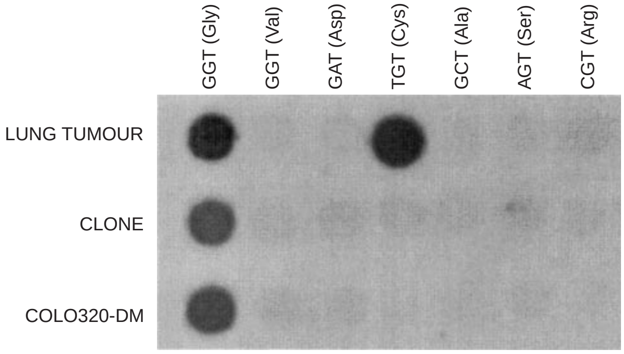

**Figure 1** Determination of point mutations in codon 12 of the K-*ras* oncogene by dot-blot hybridization analysis demonstrating that all samples are positive for wild-type probe (GGT, Gly), while a TGT (Cys) mutation is evident in one allele of the lung tumour sample. No K-*ras* mutations at codon 12 are present in the DNA of COLO320-DM cells

$IC_{50}$ value was 2.51 ± 0.11 μM. A similar value of $IC_{50}$ on *ras* farnesylation (2.68 ± 0.20 μM) was obtained by analysing the amount of isoprenylated p21*ras* protein in total cellular extracts (Figure 2D).

In addition to this, manumycin reduced the amount of the active, phosphorylated form of p42MAPK/ERK2 (Figure 3A) detected in total cellular extracts, the most important downstream effector of *ras*. When the optical density ratio of phosphorylated/non-phosphorylated p42MAPK/ERK2 bands of treated cells was analysed and compared to that of controls, it appeared that the exposure to manumycin significantly decreased the amount of the phosphorylated form of p42MAPK/ERK2, with a calculated $IC_{50}$ of 2.40 ± 0.67 μM (Figure 3B).

Manumycin did not affect the geranylgeranylation of p21*rap1* and p21*rhoA* (Figure 4), two members of the *ras* superfamily. Indeed, the presence of an upper band of slower electrophoretic motility, corresponding to the immature non-isoprenylated protein, was not detected in Western blots of total cellular extracts probed with the anti-p21*rap1* and p21*rhoA* antibodies (Figure 4).

### Absence of modification of cellular cholesterol by manumycin

The analysis of cholesterol concentration in COLO320-DM cells treated with manumycin 0.1–10 μM showed that the cholesterol: protein ratio was unchanged in manumycin-treated cells with respect to controls (Figure 5), providing further evidence that the mechanism of action of manumycin did not involve the biosynthesis of intermediates of the cholesterol biosynthetic pathway. Indeed, 3-hydroxy-3-methylglutaryl coenzyme A (HMG-CoA) reductase inhibitors, including lovastatin, decreased the overall synthesis of final products derived from the isoprenoid biosynthetic pathway, including cholesterol, dolichol and ubiquinone (Corsini et al, 1995).

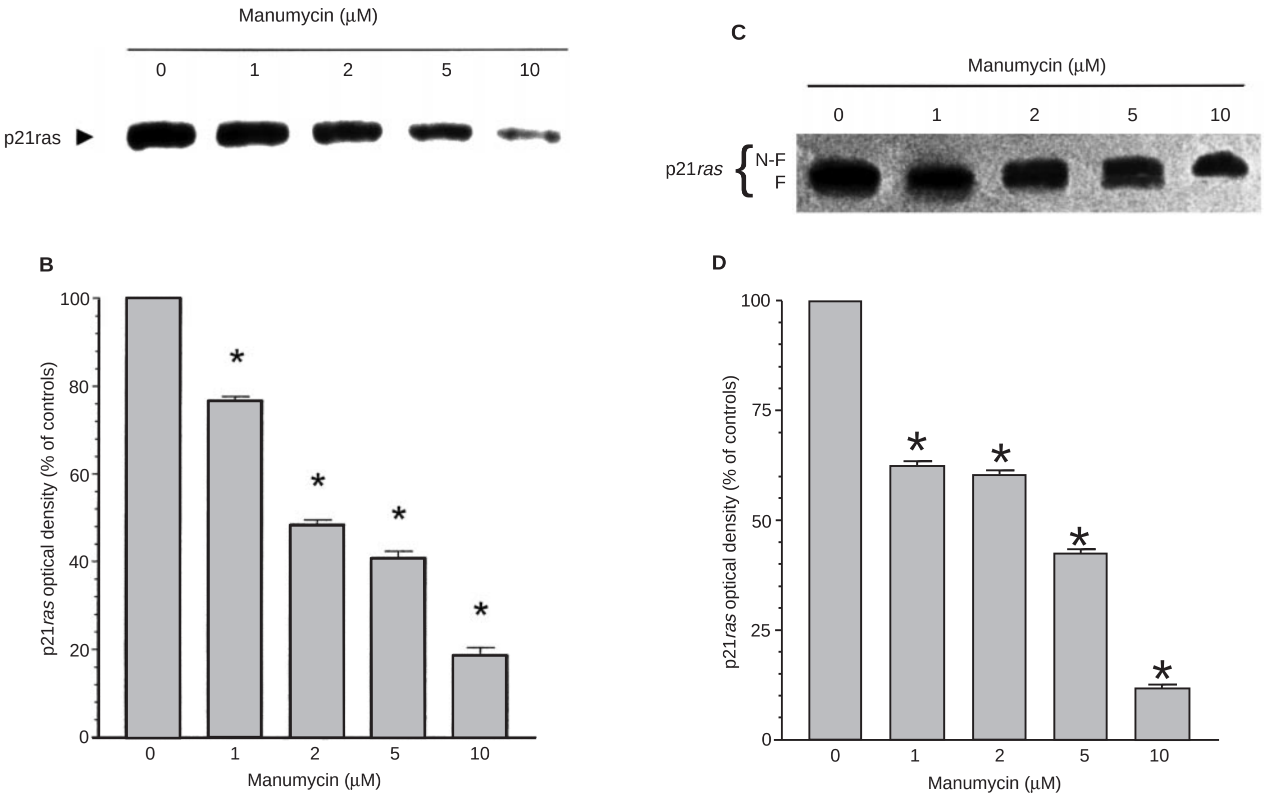

**Figure 2** Immunoblotting and image analysis of p21*ras* in samples of cellular membranes (**A, B**) and from total cellular lysates (**C, D**) of COLO320-DM cells exposed to manumycin. Columns and bars, mean values ± s.e. respectively. F, farnesylated, N-F, non-farnesylated p21*ras*; $^*P < 0.05$ vs controls

 *© 2000 Cancer Research Campaign*

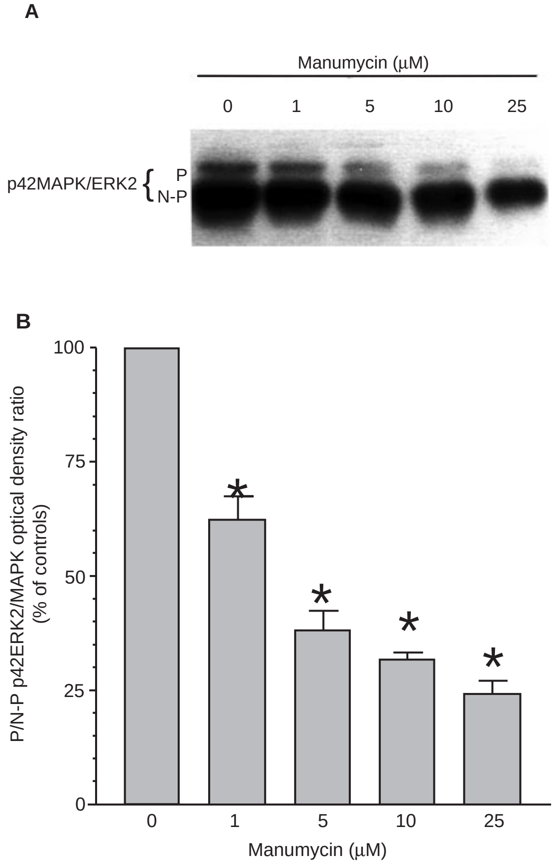

**Figure 3** Immunoblotting and image analysis of p42MAPK/ERK2 in COLO320-DM cells treated with manumycin (**A, B**). Columns and bars, mean values ± s.e. respectively. P, phosphorylated; N-P, non-phosphorylated p42MAPK/ERK2 *$P < 0.05$ vs controls

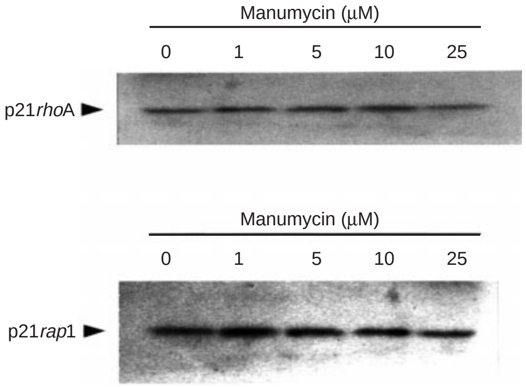

**Figure 4** Immunoblotting analysis of p21*rhoA* (upper) and p21*rap1* (lower) in COLO320-DM cells treated with manumycin

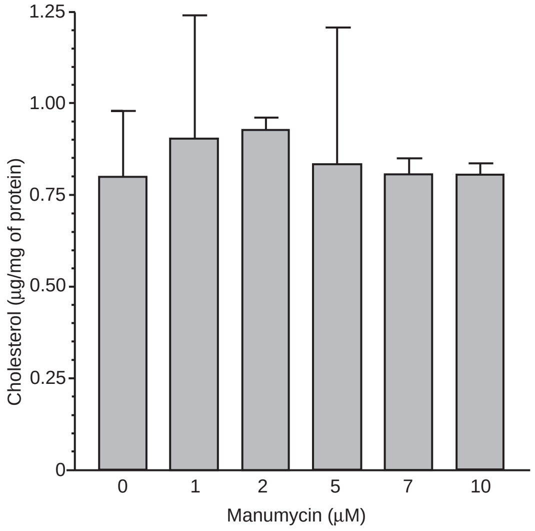

**Figure 5** Assay of total cholesterol concentration in COLO320-DM cells treated with manumycin. Columns and bars, mean values ± s.e. respectively

### Induction of apoptosis by manumycin in COLO320-DM cells

Apoptosis was demonstrated by the occurrence of DNA digestion into nucleosome-sized fragments, after extraction of DNA from cells exposed to manumycin 1–25 µM alone or in combination with MVA 100 µM (Figure 6). The extent of DNA fragmentation was dependent on both the time of exposure (Figure 6, left) and the concentration of the drug (Figure 6, middle). In particular, the presence of chromatin fragments was not clearly detectable after 24 h of treatment, but when COLO320-DM cells were exposed to the drug for longer times (48–72 h), DNA laddering became evident (Figure 6, left and middle). DNA fragmentation occurred in a dose-dependent manner in COLO320-DM cells treated with manumycin for 72 h (Figure 6, middle) and was more evident as compared to the shorter exposure. Quantitative image analysis of DNA fragmentation confirmed that the increase in drug concentrations was associated with enhanced optical density of DNA bands corresponding to shorter fragments (180–900 bp) (Figure 7A), whereas the fragmentation ratio of DNA extracted from treated cells was not proportional to drug concentration (Figure 7B). MVA was unable to reduce the apoptotic effect of manumycin (Figure 6, right), as revealed by image analysis. Indeed, the presence of MVA did not reduce the optical density of bands corresponding to shorter fragments of DNA (Figure 7A) nor the fragmentation ratio obtained with manumycin treatment (Figure 7B).

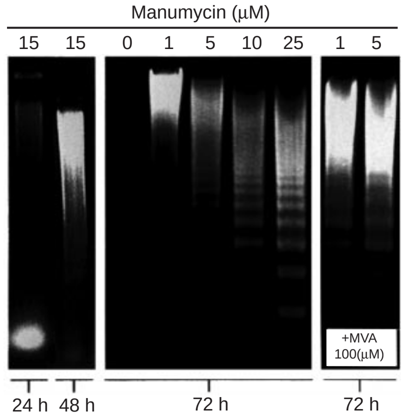

**Figure 6** Gel electrophoresis of DNA extracted from manumycin-treated COLO320-DM cells. The duration of manumycin treatment is shown on the bottom

© 2000 Cancer Research Campaign

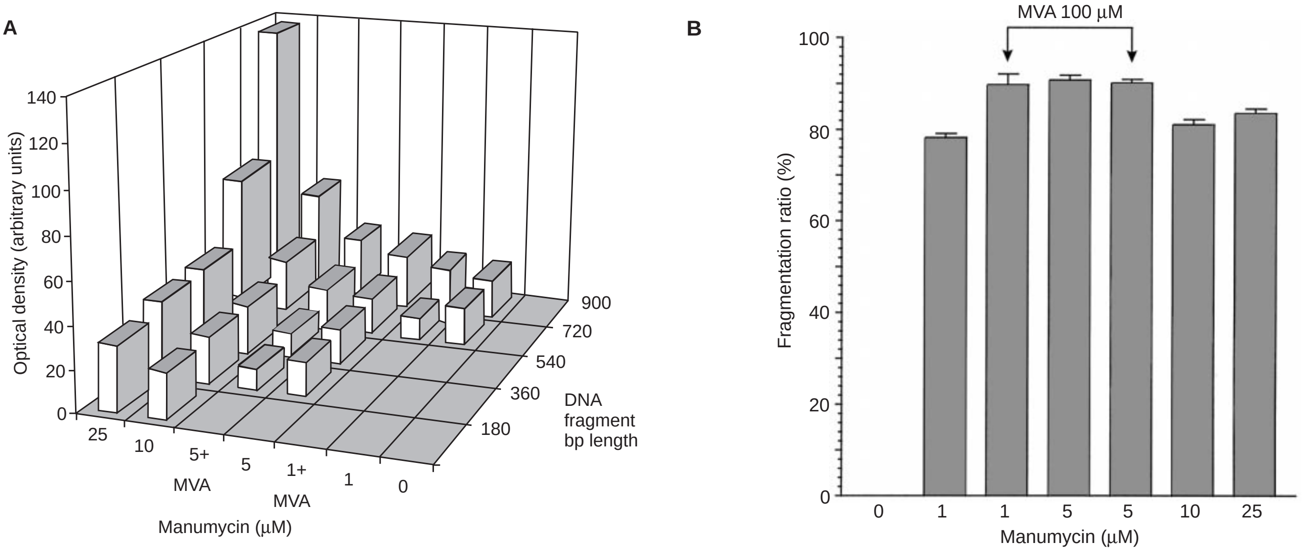

**Figure 7** Image analysis of apoptotic DNA from cells exposed to manumycin and separated by gel electrophoresis (**A**) and calculation of DNA fragmentation ratio (FR) (**B**). Columns and bars, mean values ± s.e. respectively

### Inhibition of COLO320-DM cell proliferation by manumycin

Manumycin reduced COLO320-DM cell proliferation in a dose-dependent manner (Figure 8), and the calculated $IC_{50}$ value was 3.58 ± 0.27 μM, in agreement with the results obtained in the apoptosis assays. The cytotoxic activity of manumycin 0.1–25 μM on COLO320-DM cell growth was not reduced by MVA 100 μM (Figure 8), as demonstrated by an $IC_{50}$ value (3.18 ± 0.19 μM) not significantly different from that observed in the absence of MVA ($P > 0.05$).

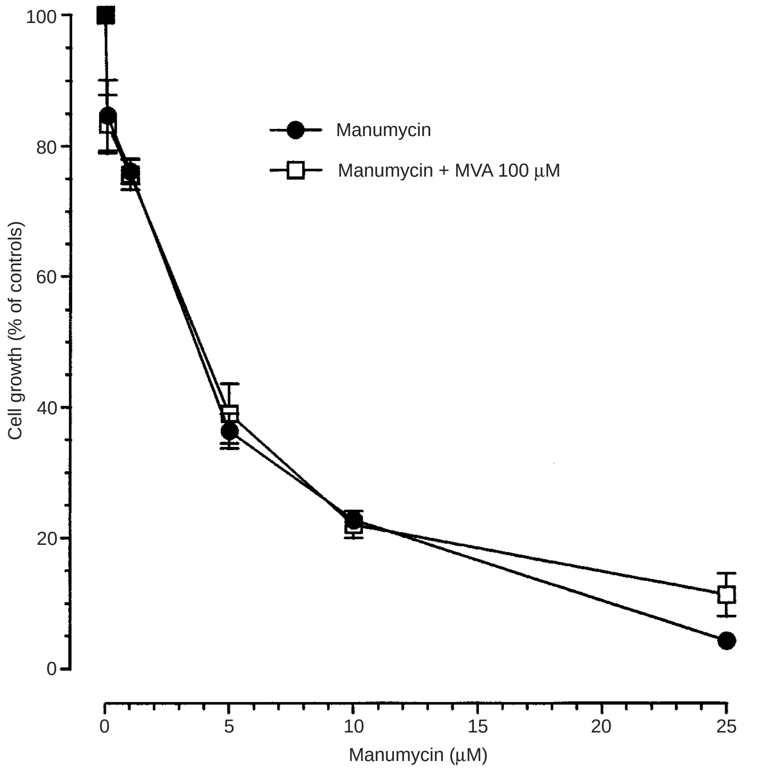

**Figure 8** Effect of manumycin alone or in combination with MVA 100 μM on COLO320-DM cell proliferation. Symbols and bars, mean values ± s.e. respectively

 © 2000 Cancer Research Campaign

### DISCUSSION

Mutated *ras* oncogenes are detected in human neoplasms, including pancreatic cancer and colorectal neoplasms (K-*ras*) and acute leukaemias (N-*ras*) (Bos, 1989). Moreover, the overexpression of *ras* oncogenes may be related to hormone-independent growth (Danesi et al, 1996), neoangiogenesis and metastasis (Larcher et al, 1996). All these biological characteristics contribute to a poor clinical prognosis and reduced survival for patients. Therefore, the aim of a number of studies was to identify new agents able to disrupt the *ras* function (Tamanoi, 1993). Among the new drugs, manumycin was first identified as an antimicrobial agent exerting a specific inhibitory action on FPTase (Hara and Han, 1995). In this view, the aim of this study was to evaluate the effect of manumycin on the growth and *ras* protein isoprenylation of a cell line derived from a human colorectal cancer expressing a normal K-*ras* gene sequence.

The effect of manumycin in cells harbouring a mutated and activated *ras* oncogene has been documented (Nagase et al, 1996). Therefore, the present study addressed the in vitro evaluation of manumycin in cells characterized by the presence of a normal *ras* gene as demonstrated by PCR analysis of K-*ras* sequence, in order to investigate whether the cytotoxicity of a FPTase inhibitor is limited to cells with a mutated *ras* gene or not. Indeed, the use of FPTase inhibitors only in tumours with a mutation of *ras* gene may be a limitation of their therapeutic usefulness since in a significant number of solid tumours K-*ras* mutation may appear late in tumour progression or does not occur.

Manumycin exerted a dose-dependent cytotoxic effect with an $IC_{50}$ value very similar to that of isolated FPTase in vitro (Tamanoi, 1993), and the inhibitory effect occurred at concentrations lower than those of inhibitors of the synthesis of farnesyl or geranylgeranyl moieties, including lovastatin (Corsini et al, 1995). Furthermore, the present study demonstrates that MVA, from which the isoprenyl donors farnesyl-pyrophosphate and geranylgeranyl-pyrophosphate are synthesized (Maltese, 1990), was not able to reduce the cytotoxic effect of manumycin, suggesting that the increased availability of isoprenoid moieties does not overcome the inhibition of FPTase by manumycin.

It is interesting to note that the inhibitory effect of manumycin on the HepG2 cell line, harbouring a mutated, activated *ras* oncogene (Nagase et al, 1996), occurred at higher concentrations than those required for COLO320-DM, that expresses a normal K-*ras* gene as reported in the present study. These discrepancies in manumycin sensitivity among cell lines may be due to different levels of expression or activity of *ras*, thus requiring a wide range of manumycin concentrations to inhibit *ras* isoprenylation. The data of the present study demonstrate that blocking the function of a normal *ras* gene results in cell death, thus providing evidences that cancer cells rely on the *ras* pathway whether the gene product is normal or mutated.

The results from Western blot assays strenghtened the evidence that the cytotoxic effect of manumycin could be dependent on the inhibition of p21*ras* protein farnesylation. In addition to this, the signal transduction from membrane receptors to downstream effectors of p21*ras* was interrupted, as demonstrated by the evaluation of p42MAPK/ERK2 activation. Indeed, COLO320-DM cells, exposed to manumycin, showed a dose-dependent decrease in the amount of activated, phosphorylated p42MAPK/ERK2, a cytoplasmatic serine/threonine protein-kinase that plays a fundamental role in the transduction of a number of mitogenic signals, including growth factors (Marshall, 1995). In particular, p42MAPK/ERK2 seems to be the major cytoplasmic effector of p21*ras*-GTP activated complex, and the increase in the phosphorylated, active form of p42MAPK/ERK2 is associated with gene transcription and cellular proliferation (Marshall, 1996). Therefore, the reduction of p21*ras* farnesylation caused a decrease in p42MAPK/ERK2 activation, thus interfering with the signal transduction from the cell membrane to the intracellular effectors via the p21*ras*-p42MAPK/ERK2 pathway. The $IC_{50}$ values of p21*ras* and p42MAPK/ERK2 inhibition as well as the reduction of cell viability appeared to be similar, providing evidence about the dependence of one phenomenon from another.

In the present study, p21*rhoA* and p21*rap1* were monitored to study whether protein geranylgeranylation was affected by manumycin or its effect was specific on farnesylation. These proteins undergo geranylgeranylation by the geranylgeranyl: protein transferase I; as a matter of fact, the results demonstrated that their post-translational modification was not affected by manumycin. The p21*rhoA* protein has a role in the regulation of focal adhesion processes, differentiation and signal transduction via integrins (Giancotti and Mainiero, 1994), while p21*rap1* protein is physiological inhibitor of p21*ras* function (Ikeda et al, 1995; Nassar et al, 1995; Wittinghofer and Herrman, 1995). Therefore, the prevalence of p21*rap1* function over the inhibited p21*ras* may be an additional factor contributing to the overall activity of manumycin.

The importance of inhibition of Gy subunits and nuclear laminin farnesylation (Giannakouros and Magee, 1993) by manumycin cannot be underestimated, as well as the prenylation of other proteins which required farnesyl moieties for their function (Cox and Der, 1997). However, it appears that the inhibition of p21*ras* farnesylation is a determinant factor in the antiproliferative effect of manumycin on COLO320-DM cells. Apoptosis is an energy-dependent process of cell death characterized by morphological and biochemical alterations. One of the hallmarks of the apoptotic death is the internucleosomal DNA fragmentation, with production and release of oligonucleosomal size DNA fragments in the cytoplasm (Kawabata et al, 1994). The occurrence of this phenomenon during treatment with isoprenylated inhibitors, like manumycin, is explained by the fact that many prenylated proteins, including laminins A (Gibbs and Oliff, 1997), are involved in the regulation and integrity of cell structures, such as the nuclear envelope and lysosomes (Maltese, 1990; Perez-Sala and Mollinedo, 1994), with possible loss of enzyme compartmentalization and DNA fragmentation (Danesi et al, 1996). It can be concluded that the inhibition of FPTase activity by manumycin in COLO320-DM cells is an important factor for DNA degradation.

Inhibitors of HMG-CoA, including lovastatin, prevent isoprenylation by reducing the synthesis of farnesyl and geranylgeranyl moieties, but also the final products (cholesterol, dolichol, ubiquinone) of MVA pathway (Corsini et al, 1995). Therefore, the clinical use of statins may be characterized by the occurrence of adverse effects, such as rhabdomiolysis and peripheral neuropathy (Corsini et al, 1995; Phan et al, 1995; Veerkamp et al, 1996). On the contrary, manumycin is a specific FPTase inhibitor that does not affect the biosynthesis of end products of isoprenoid metabolism.

In conclusion, the results of the present study demonstrate that manumycin is cytotoxic and induces apoptosis in a human cell line

© 2000 Cancer Research Campaign derived from a colorectal cancer which expresses a wild-type K-*ras* gene. This effect is obtained by inhibition of p21*ras* farnesylation that reduces signal transduction through the p21*ras*-p42MAPK/ERK2 pathway, without affecting protein geranylgeranylation or the synthesis of final products of the MVA biosynthetic pathway.

### ACKNOWLEDGEMENTS

The authors wish to thank Dr M Hara for the insightful discussion of the data presented in this paper.

### REFERENCES

Bos JL (1989) Ras oncogenes in human cancer: a review. *Cancer Res* **49:** 4682–4689

Corsini A, Maggi FM and Catapano AL (1995) Pharmacology of competitive inhibitors of HMG-CoA reductase. *Pharmacol Res* **31**: 9–27

Cox AD and Der CJ (1997) Farnesyltransferase inhibitors and cancer treatment: targeting simply Ras? *Biochim Biophys Acta* **1333**: F51–F71

Danesi R, Figg WD, Reed E and Myers CE (1995) Paclitaxel (Taxol®) inhibits protein isoprenylation and induces apoptosis in PC-3 human prostate cancer cells. *Mol Pharmacol* **47**: 1106–1111

Danesi R, Nardini D, Basolo F, Del Tacca M, Samid D and Myers CE (1996) Phenylacetate inhibits protein isoprenylation and growth of the androgen-independent LNCaP prostate cancer cells transfected with the T24 Ha-ras oncogene. *Mol Pharmacol* **49**: 972–979

Giancotti FG and Mainiero F (1994) Integrin-mediated adhesion and signaling in tumorigenesis. *Biochim Biophys Acta* **1198**: 47–64

Giannakouros T and Magee AI (1993) Protein prenylation and associated modifications. In: *Lipid Modifications of Proteins*, Schlesinger MJ (ed), pp. 136–162. CRC Press; Boca Raton, FL

Gibbs JB and Oliff A (1997) The potential of farnesyltransferase inhibitors as cancer chemotherapeutics. *Annu Rev Pharmacol Toxicol* **37**: 143–146

Gibbs JB, Oliff A and Kohl NE (1994) Farnesyltransferase inhibitors: *ras* research yields a potential cancer therapeutics. *Cell* **77**: 175–178

Hara M and Han M (1995) *Ras* farnesyltransferase inhibitors suppress the phenotype resulting from an activated ras mutation in *Caenorhabditis elegans*. *Proc Natl Acad Sci USA* **92**: 3333–3337

Hara M, Kazuhito A, Akinaga S, Okabe M, Nakano H, Gomez R, Wood D, Uh M and Tamanoi F (1993) Identification of ras farnesyltransferase inhibitors by microbial screening. *Proc Natl Acad Sci USA* **90**: 2281–2285

Ikeda M, Koyama S, Okazaki M, Dohi K and Kikuchi A (1995) Rap1 p21 regulates the interaction of *ras* p21 with RGL, a new effector protein of *ras* p21. *FEBS Lett* **375**: 37–40

Kawabata H, Anzai N, Yoshida Y and Okuma M (1994) A new method for quantitative estimation of the degree of DNA fragmentation utilizing agarose gel electrophoresis. *Int J Hematol* **59**: 311–316

Larcher F, Robles AI, Duran H, Murillas R, Quintanilla M, Cano A, Conti CJ and Jorcano JL (1996) Up-regulation of vascular endothelial growth factor/vascular permeability factor in mouse skin carcinogenesis correlates with malignant progression state and activated H-*ras* expression levels. *Cancer Res* **56**: 5391–5396

Levitzky A (1996) Targeting signal transduction for disease therapy. *Curr Opin Cell Biol* **8**: 239–244

Lowry OH, Rosebrough NJ, Farr AL and Randall RJ (1951) Protein measurement with folin phenol reagent. *J Biol Chem* **193**: 265–267

Lowy DR and Willumsen BM (1993) Function and regulation of *ras*. *Annu Rev Biochem* **62**: 851–891

Maltese WA (1990) Post-translational modification of proteins by isoprenoids in mammalian cells. *FASEB J* **4**: 3319–3328

Marchetti A, Buttitta F, Pellegrini S, Chella A, Bertacca G, Filardo A, Tognoni V, Ferreli F, Signorini E, Angeletti CA and Bevilacqua G (1996) Bronchioloalveolar lung carcinomas: K-*ras* mutations constant events in the mucinous subtype. *J Pathol* **179**: 254–259

Marshall CJ (1993) Protein prenylation: a mediator of protein–protein interactions. *Science* **259**: 1865–1866

Marshall CJ (1996) Ras effectors. *Curr Opin Cell Biol* **8**: 197–204

Marshall MS (1995) Ras target proteins in eukaryotic cells. *FASEB J* **9**: 1311–1318

Nagase T, Kawata S, Tamura S, Matsuda Y, Inui Y, Yamasaky E, Ishiguro H, Ito T and Matsuzawa Y (1996) Inhibition of cell growth of human hepatoma cell line (Hep G2) by a farnesyl protein transferase inhibitor: a preferential suppression of *ras* farnesylation. *Int J Cancer* **65**: 620–626

Nagasu T, Yoshimatsu K, Rowell C, Lewis MD and Garcia AM (1995) Inhibition of human tumor xenograft growth by treatment with farnesyl transferase inhibitor B956. *Cancer Res* **55**: 5310–5314

Nassar N, Horn G, Hermann C, Scherer A, McCormick F and Wittinghofer A (1995) The 2.2 Å crystal structure of the Ras-binding domain of the serine/threonine kinase c-Raf1 in complex with Rap1A and a GTP analogue. *Nature* **375**: 554–560

Perez-Sala D and Mollinedo F (1994) Inhibition of isoprenoid biosynthesis induces apoptosis in human promyelocytic HL-60 cells. *Biochem Biophys Res Commun* **199**: 1209–1215

Phan T, McLeod JG, Pollard JD, Peiris O, Rohan A and Halpern J-P (1995) Peripheral neuropathy associated with simvastatin. *J Neurol Neurosurg Psychiatry* **58**: 625–628

Rodenhuis S, Boerrigter L, Top B, Slebos RJC, Mooi WJ, van't Veer L and van Zandwijk N (1997) Mutational activation of the K-*ras* oncogene and the effect of chemotherapy in advanced adenocarcinoma of the lung: a prospective study. *J Clin Oncol* **15**: 285–291

Tamanoi F (1993) Inhibitors of *Ras* farnesyltransferases. *Trends Biol Sci* **18**: 349–353

Veerkamp JH, Smith JWA, Benders AAGM and Oosterhof A (1996) Effects of HMG-CoA reductase inhibitors on growth and differentiation of cultured rat skeletal muscle cells. *Biochim Biophys Acta* **1315**: 217–223

Wittinghofer A and Herrman C (1995) *Ras*-effector interactions, the problem of specificity. *FEBS Lett* **369**: 52–56

*© 2000 Cancer Research Campaign*
